# *N*′-(2-Hy­droxy-1-naphth­yl­methylidene)-3-meth­oxy­benzohydrazide

**DOI:** 10.1107/S1600536810022026

**Published:** 2010-06-16

**Authors:** Zhi-Xi Hang

**Affiliations:** a College of Biological and Chemical Engineering, Anhui Polytechnic University, Wuhu 241000, People’s Republic of China

## Abstract

In the title compound, C_19_H_16_N_2_O_3_, the dihedral angle between the naphthalene ring system and the benzene ring is 19.8 (3)°. An intra­molecular O—H⋯N hydrogen bond stabilizes the mol­ecular conformation. In the crystal, mol­ecules are linked *via* inter­molecular N—H⋯O hydrogen bonds, forming chains along the *a* axis.

## Related literature

For the biological activity of hydrazone compounds, see: Arunkumar *et al.* (2006 ▶); Saxena *et al.* (2008 ▶); Zia-ur-Rehman *et al.* (2009 ▶); Galal *et al.* (2009 ▶); Bordoloi *et al.* (2009 ▶). For similar hydrazone compounds, see: Han *et al.* (2010 ▶); Wang *et al.* (2010 ▶); Qiao *et al.* (2010 ▶); Suleiman Gwaram *et al.* (2010 ▶); Sun *et al.* (2009 ▶).
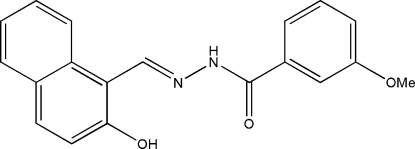


## Experimental

### 

#### Crystal data

C_19_H_16_N_2_O_3_
*M* *_r_* = 320.34
Monoclinic, 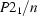

*a* = 7.1700 (15) Å
*b* = 31.174 (7) Å
*c* = 7.4669 (16) Å
β = 109.746 (12)°
*V* = 1570.9 (6) Å^3^
*Z* = 4
Mo *K*α radiation
μ = 0.09 mm^−1^
*T* = 298 K
0.18 × 0.17 × 0.17 mm

#### Data collection

Bruker SMART CCD area-detector diffractometer
Absorption correction: multi-scan (*SADABS*; Sheldrick, 1996 ▶) *T*_min_ = 0.984, *T*_max_ = 0.984
9232 measured reflections
3405 independent reflections
1839 reflections with *I* > 2σ(*I*)
*R*_int_ = 0.093

#### Refinement

*R*[*F* ^2^ > 2σ(*F* ^2^)] = 0.048
*wR*(*F* ^2^) = 0.141
*S* = 0.92
3405 reflections
222 parameters
1 restraint
H atoms treated by a mixture of independent and constrained refinement
Δρ_max_ = 0.22 e Å^−3^
Δρ_min_ = −0.23 e Å^−3^

### 

Data collection: *SMART* (Bruker, 2002 ▶); cell refinement: *SAINT* (Bruker, 2002 ▶); data reduction: *SAINT*; program(s) used to solve structure: *SHELXS97* (Sheldrick, 2008 ▶); program(s) used to refine structure: *SHELXL97* (Sheldrick, 2008 ▶); molecular graphics: *SHELXTL* (Sheldrick, 2008 ▶); software used to prepare material for publication: *SHELXL97*.

## Supplementary Material

Crystal structure: contains datablocks global, I. DOI: 10.1107/S1600536810022026/rz2461sup1.cif

Structure factors: contains datablocks I. DOI: 10.1107/S1600536810022026/rz2461Isup2.hkl

Additional supplementary materials:  crystallographic information; 3D view; checkCIF report

## Figures and Tables

**Table 1:** Hydrogen-bond geometry (Å, °)

*D*—H⋯*A*	*D*—H	H⋯*A*	*D*⋯*A*	*D*—H⋯*A*
N2—H2⋯O2^i^	0.91 (1)	1.97 (1)	2.842 (3)	163 (2)
O1—H1⋯N1	0.82	1.85	2.574 (2)	146

Symmetry code: (i) 
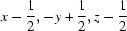
.

**Fig. 1.**
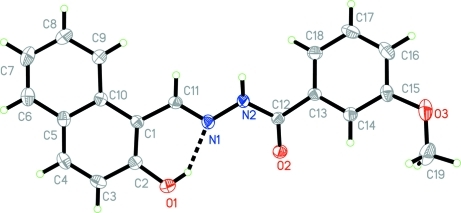
Anisotropic displacement ellipsoid plot of the title compound at the 30% probability level. Hydrogen atoms are drawn as spheres of arbitrary radius. The intramolecular O–H···N hydrogen bond is drawn as a dashed line.

**Fig. 2.**
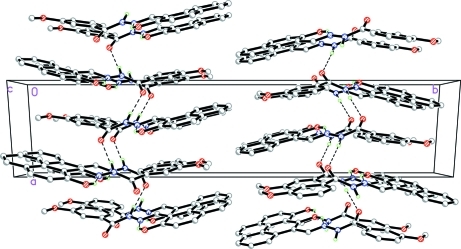
Packing diagram of the title compound viewed along the c axis. Intermolecular hydrogen bonds are shown as dashed lines.

## supplementary crystallographic information

### Comment

Considerable interest has been focused on hydrazone compounds due to their excellent biological activities (Arunkumar *et al.*, 2006; Saxena *et al.*, 2008; Zia-ur-Rehman *et al.*, 2009; Galal *et al.*, 2009; Bordoloi *et al.*, 2009). In the last few years, a great deal of hydrazone compounds have been prepared and characterized by X-ray diffraction (Han *et al.*, 2010; Wang *et al.*, 2010; Qiao *et al.*, 2010; Suleiman Gwaram *et al.*, 2010; Sun *et al.*, 2009). The present paper reports a new hydrazone compound, *N'*-(2-hydroxynaphthylene)-3-methoxybenzohydrazide.

In the title compound (Fig. 1) the dihedral angle between the naphthalene ring system and the benzene ring is 19.8 (3)°. Bond lengths and angles are comparable to those found in similar hydrazone compounds cited above. An intramolecular O—H···N hydrogen bond (Table 1) stabilizes the molecular conformation. The molecules are linked *via* intermolecular N—H···O hydrogen bonds (Table 1), to form chains along the *a* axis (Fig. 2).

### Experimental

Equimolar quantities (1 mmol) of 3-methoxybenzohydrazide and 2-hydroxy-1-naphthyaldehyde were mixed and stirred in methanol for 2 h at ambient temperature. The resulting mixture was concentrated under recuced pressure. The residue, purified by washing with cold methanol and diethyl ether, afforded the pure product of the hydrazone compound. Colourless single crystals suitable for X-ray diffraction were obtained on slow evaqporation of a methanol solution.

### Refinement

The H2 atom was found from a difference Fourier map and refined with an isotropic displacement parameter of 0.08 Å^2^, and with the N–H distance restrained to 0.90 (1) Å. The remaining H atoms were positioned geometrically and refined using a riding model, with C–H = 0.93 and 0.96 Å, O–H = 0.82 Å, and *U*_iso_(H) = 1.2 or 1.5*U*_eq_(C, O).

### Crystal data

**Table:**

C_19_H_16_N_2_O_3_	*F*(000) = 672
*M**_r_* = 320.34	*D*_x_ = 1.355 Mg m^−^^3^
Monoclinic, *P*2_1_/*n*	Mo *K*α radiation, λ = 0.71073 Å
Hall symbol: -P 2yn	Cell parameters from 1460 reflections
*a* = 7.1700 (15) Å	θ = 2.5–24.0°
*b* = 31.174 (7) Å	µ = 0.09 mm^−^^1^
*c* = 7.4669 (16) Å	*T* = 298 K
β = 109.746 (12)°	Block, colourless
*V* = 1570.9 (6) Å^3^	0.18 × 0.17 × 0.17 mm
*Z* = 4	

### Data collection

**Table:**

Bruker SMART CCD area-detector diffractometer	3405 independent reflections
Radiation source: fine-focus sealed tube	1839 reflections with *I* > 2σ(*I*)
graphite	*R*_int_ = 0.093
ω scans	θ_max_ = 27.0°, θ_min_ = 1.3°
Absorption correction: multi-scan (*SADABS*; Sheldrick, 1996)	*h* = −9→9
*T*_min_ = 0.984, *T*_max_ = 0.984	*k* = −39→35
9232 measured reflections	*l* = −7→9

### Refinement

**Table:**

Refinement on *F*^2^	Primary atom site location: structure-invariant direct methods
Least-squares matrix: full	Secondary atom site location: difference Fourier map
*R*[*F*^2^ > 2σ(*F*^2^)] = 0.048	Hydrogen site location: inferred from neighbouring sites
*wR*(*F*^2^) = 0.141	H atoms treated by a mixture of independent and constrained refinement
*S* = 0.92	*w* = 1/[σ^2^(*F*_o_^2^) + (0.0597*P*)^2^] where *P* = (*F*_o_^2^ + 2*F*_c_^2^)/3
3405 reflections	(Δ/σ)_max_ < 0.001
222 parameters	Δρ_max_ = 0.22 e Å^−^^3^
1 restraint	Δρ_min_ = −0.23 e Å^−^^3^

### Special details

**Table:**

Geometry. All e.s.d.'s (except the e.s.d. in the dihedral angle between two l.s. planes) are estimated using the full covariance matrix. The cell e.s.d.'s are taken into account individually in the estimation of e.s.d.'s in distances, angles and torsion angles; correlations between e.s.d.'s in cell parameters are only used when they are defined by crystal symmetry. An approximate (isotropic) treatment of cell e.s.d.'s is used for estimating e.s.d.'s involving l.s. planes.
Refinement. Refinement of *F*^2^ against ALL reflections. The weighted *R*-factor *wR* and goodness of fit *S* are based on *F*^2^, conventional *R*-factors *R* are based on *F*, with *F* set to zero for negative *F*^2^. The threshold expression of *F*^2^ > σ(*F*^2^) is used only for calculating *R*-factors(gt) *etc*. and is not relevant to the choice of reflections for refinement. *R*-factors based on *F*^2^ are statistically about twice as large as those based on *F*, and *R*- factors based on ALL data will be even larger.

### Fractional atomic coordinates and isotropic or equivalent isotropic displacement parameters (Å^2^)

**Table:**

	*x*	*y*	*z*	*U*_iso_*/*U*_eq_	
N1	0.9564 (3)	0.21280 (5)	0.6283 (3)	0.0368 (5)	
N2	0.9265 (3)	0.24056 (6)	0.4765 (3)	0.0377 (5)	
O1	1.0227 (3)	0.19610 (5)	0.9820 (2)	0.0522 (5)	
H1	1.0193	0.2108	0.8900	0.078*	
O2	1.1086 (2)	0.29306 (4)	0.6668 (2)	0.0446 (4)	
O3	0.8581 (3)	0.42263 (5)	0.2180 (3)	0.0610 (5)	
C1	0.9067 (3)	0.14315 (6)	0.7351 (3)	0.0325 (5)	
C2	0.9652 (3)	0.15572 (7)	0.9238 (3)	0.0374 (5)	
C3	0.9684 (4)	0.12616 (8)	1.0675 (3)	0.0448 (6)	
H3	1.0045	0.1353	1.1932	0.054*	
C4	0.9194 (4)	0.08463 (8)	1.0244 (4)	0.0465 (6)	
H4	0.9231	0.0656	1.1215	0.056*	
C5	0.8625 (3)	0.06948 (7)	0.8351 (3)	0.0385 (6)	
C6	0.8142 (4)	0.02588 (8)	0.7898 (4)	0.0512 (7)	
H6	0.8181	0.0068	0.8867	0.061*	
C7	0.7624 (4)	0.01126 (8)	0.6091 (5)	0.0632 (8)	
H7	0.7330	−0.0176	0.5820	0.076*	
C8	0.7539 (5)	0.04030 (8)	0.4638 (4)	0.0667 (8)	
H8	0.7171	0.0305	0.3390	0.080*	
C9	0.7981 (4)	0.08248 (8)	0.5010 (4)	0.0521 (7)	
H9	0.7913	0.1009	0.4010	0.063*	
C10	0.8543 (3)	0.09898 (7)	0.6881 (3)	0.0351 (5)	
C11	0.8922 (3)	0.17435 (7)	0.5871 (3)	0.0358 (5)	
H11	0.8354	0.1664	0.4601	0.043*	
C12	1.0051 (3)	0.28023 (7)	0.5079 (3)	0.0330 (5)	
C13	0.9564 (3)	0.30824 (7)	0.3373 (3)	0.0324 (5)	
C14	0.9349 (3)	0.35181 (7)	0.3628 (3)	0.0363 (5)	
H14	0.9528	0.3624	0.4839	0.044*	
C15	0.8871 (3)	0.37955 (7)	0.2097 (4)	0.0417 (6)	
C16	0.8640 (4)	0.36340 (9)	0.0300 (4)	0.0530 (7)	
H16	0.8325	0.3819	−0.0736	0.064*	
C17	0.8871 (4)	0.32039 (9)	0.0040 (4)	0.0535 (7)	
H17	0.8726	0.3100	−0.1167	0.064*	
C18	0.9320 (3)	0.29230 (7)	0.1567 (3)	0.0417 (6)	
H18	0.9457	0.2631	0.1387	0.050*	
C19	0.8793 (5)	0.44027 (8)	0.3982 (5)	0.0690 (9)	
H19A	0.7877	0.4266	0.4486	0.104*	
H19B	0.8525	0.4705	0.3853	0.104*	
H19C	1.0122	0.4357	0.4830	0.104*	
H2	0.836 (3)	0.2332 (9)	0.363 (2)	0.080*	

### Atomic displacement parameters (Å^2^)

**Table:**

	*U*^11^	*U*^22^	*U*^33^	*U*^12^	*U*^13^	*U*^23^
N1	0.0382 (11)	0.0332 (10)	0.0349 (11)	0.0007 (9)	0.0070 (9)	0.0046 (9)
N2	0.0449 (12)	0.0303 (10)	0.0301 (11)	−0.0025 (9)	0.0024 (9)	0.0034 (9)
O1	0.0689 (12)	0.0450 (10)	0.0397 (10)	−0.0084 (10)	0.0143 (10)	−0.0079 (8)
O2	0.0511 (10)	0.0396 (9)	0.0309 (9)	−0.0023 (8)	−0.0020 (8)	−0.0008 (7)
O3	0.0633 (13)	0.0403 (10)	0.0735 (14)	0.0055 (9)	0.0154 (11)	0.0180 (10)
C1	0.0297 (12)	0.0359 (12)	0.0311 (13)	0.0015 (10)	0.0091 (10)	0.0030 (10)
C2	0.0353 (13)	0.0369 (13)	0.0390 (14)	0.0017 (11)	0.0114 (11)	−0.0016 (11)
C3	0.0468 (15)	0.0558 (16)	0.0301 (13)	0.0031 (13)	0.0108 (12)	0.0026 (12)
C4	0.0460 (15)	0.0502 (15)	0.0443 (15)	0.0037 (13)	0.0167 (12)	0.0151 (13)
C5	0.0344 (13)	0.0353 (12)	0.0469 (15)	0.0032 (11)	0.0150 (11)	0.0058 (11)
C6	0.0495 (16)	0.0413 (14)	0.0623 (18)	0.0014 (13)	0.0184 (14)	0.0107 (14)
C7	0.071 (2)	0.0338 (14)	0.082 (2)	−0.0066 (13)	0.0229 (18)	−0.0013 (15)
C8	0.093 (2)	0.0472 (16)	0.0570 (19)	−0.0146 (16)	0.0211 (18)	−0.0146 (15)
C9	0.0714 (19)	0.0417 (14)	0.0436 (15)	−0.0093 (13)	0.0199 (14)	−0.0033 (12)
C10	0.0328 (12)	0.0344 (12)	0.0371 (13)	0.0016 (10)	0.0105 (11)	0.0010 (11)
C11	0.0370 (13)	0.0371 (12)	0.0296 (12)	0.0000 (11)	0.0065 (10)	−0.0002 (10)
C12	0.0326 (12)	0.0318 (12)	0.0305 (12)	0.0020 (10)	0.0054 (10)	−0.0011 (10)
C13	0.0285 (11)	0.0363 (12)	0.0282 (12)	−0.0034 (10)	0.0042 (9)	−0.0006 (10)
C14	0.0327 (12)	0.0370 (12)	0.0368 (13)	−0.0025 (10)	0.0084 (10)	−0.0009 (11)
C15	0.0333 (13)	0.0387 (13)	0.0481 (15)	−0.0008 (11)	0.0073 (11)	0.0112 (12)
C16	0.0485 (16)	0.0640 (18)	0.0408 (16)	−0.0083 (14)	0.0075 (12)	0.0168 (14)
C17	0.0556 (17)	0.0742 (19)	0.0307 (14)	−0.0132 (15)	0.0147 (12)	−0.0008 (14)
C18	0.0440 (14)	0.0447 (13)	0.0358 (14)	−0.0041 (12)	0.0126 (11)	−0.0034 (11)
C19	0.074 (2)	0.0426 (15)	0.100 (3)	0.0077 (15)	0.042 (2)	0.0013 (16)

### Geometric parameters (Å, °)

**Table:**

N1—C11	1.284 (3)	C7—C8	1.398 (4)
N1—N2	1.384 (2)	C7—H7	0.9300
N2—C12	1.346 (3)	C8—C9	1.359 (3)
N2—H2	0.905 (10)	C8—H8	0.9300
O1—C2	1.350 (2)	C9—C10	1.414 (3)
O1—H1	0.8200	C9—H9	0.9300
O2—C12	1.235 (3)	C11—H11	0.9300
O3—C15	1.364 (3)	C12—C13	1.485 (3)
O3—C19	1.413 (3)	C13—C14	1.387 (3)
C1—C2	1.384 (3)	C13—C18	1.391 (3)
C1—C10	1.439 (3)	C14—C15	1.381 (3)
C1—C11	1.450 (3)	C14—H14	0.9300
C2—C3	1.408 (3)	C15—C16	1.390 (3)
C3—C4	1.351 (3)	C16—C17	1.373 (3)
C3—H3	0.9300	C16—H16	0.9300
C4—C5	1.414 (3)	C17—C18	1.387 (3)
C4—H4	0.9300	C17—H17	0.9300
C5—C6	1.415 (3)	C18—H18	0.9300
C5—C10	1.418 (3)	C19—H19A	0.9600
C6—C7	1.351 (4)	C19—H19B	0.9600
C6—H6	0.9300	C19—H19C	0.9600
			
C11—N1—N2	116.33 (18)	C9—C10—C5	116.9 (2)
C12—N2—N1	119.46 (18)	C9—C10—C1	123.6 (2)
C12—N2—H2	121.4 (18)	C5—C10—C1	119.5 (2)
N1—N2—H2	118.1 (18)	N1—C11—C1	121.1 (2)
C2—O1—H1	109.5	N1—C11—H11	119.5
C15—O3—C19	117.2 (2)	C1—C11—H11	119.5
C2—C1—C10	118.92 (19)	O2—C12—N2	123.04 (19)
C2—C1—C11	120.4 (2)	O2—C12—C13	121.64 (19)
C10—C1—C11	120.69 (19)	N2—C12—C13	115.32 (19)
O1—C2—C1	123.1 (2)	C14—C13—C18	120.0 (2)
O1—C2—C3	116.1 (2)	C14—C13—C12	117.57 (19)
C1—C2—C3	120.8 (2)	C18—C13—C12	122.48 (19)
C4—C3—C2	120.6 (2)	C15—C14—C13	120.5 (2)
C4—C3—H3	119.7	C15—C14—H14	119.7
C2—C3—H3	119.7	C13—C14—H14	119.7
C3—C4—C5	121.5 (2)	O3—C15—C14	125.3 (2)
C3—C4—H4	119.3	O3—C15—C16	115.5 (2)
C5—C4—H4	119.3	C14—C15—C16	119.2 (2)
C4—C5—C6	121.6 (2)	C17—C16—C15	120.6 (2)
C4—C5—C10	118.7 (2)	C17—C16—H16	119.7
C6—C5—C10	119.7 (2)	C15—C16—H16	119.7
C7—C6—C5	121.7 (2)	C16—C17—C18	120.4 (2)
C7—C6—H6	119.2	C16—C17—H17	119.8
C5—C6—H6	119.2	C18—C17—H17	119.8
C6—C7—C8	118.8 (2)	C17—C18—C13	119.3 (2)
C6—C7—H7	120.6	C17—C18—H18	120.4
C8—C7—H7	120.6	C13—C18—H18	120.4
C9—C8—C7	121.5 (3)	O3—C19—H19A	109.5
C9—C8—H8	119.3	O3—C19—H19B	109.5
C7—C8—H8	119.3	H19A—C19—H19B	109.5
C8—C9—C10	121.5 (2)	O3—C19—H19C	109.5
C8—C9—H9	119.2	H19A—C19—H19C	109.5
C10—C9—H9	119.2	H19B—C19—H19C	109.5

### Hydrogen-bond geometry (Å, °)

**Table:**

*D*—H···*A*	*D*—H	H···*A*	*D*···*A*	*D*—H···*A*
N2—H2···O2^i^	0.91 (1)	1.97 (1)	2.842 (3)	163 (2)
O1—H1···N1	0.82	1.85	2.574 (2)	146

Symmetry codes: (i) *x*−1/2, −*y*+1/2, *z*−1/2.
